# Modulatory Role of ATG5 Protein in Immune Modulation During Experimental Tularemia

**DOI:** 10.3390/microorganisms14071593

**Published:** 2026-07-21

**Authors:** Mirna Mihelčić, Ina Viduka, Maša Antonić, Andreja Zubković, Valentina Marečić, Mateja Ožanič, Kjell Eneslätt, Maja Abram, Anders Sjöstedt, Marina Šantić

**Affiliations:** 1 Department of Microbiology and Parasitology, Faculty of Medicine, University of Rijeka, 51 000 Rijeka, Croatia; mirna.mihelcic@medri.uniri.hr (M.M.); ina.viduka@medri.uniri.hr (I.V.); masa.antonic@medri.uniri.hr (M.A.); andrejaz@medri.uniri.hr (A.Z.); maja.abram@medri.uniri.hr (M.A.); 2 Department of Clinical Microbiology, Umeå University, SE-901 87 Umeå, Sweden; kjell.eneslatt@umu.se (K.E.); anders.sjostedt@umu.se (A.S.); 3 Department of Environmental Health, Teaching Institute of Public Health of Primorje-Gorski Kotar County, 51 000 Rijeka, Croatia

**Keywords:** ATG5, autophagy, *Francisella*, IL-1β, immunity, mice

## Abstract

Autophagy is a crucial mechanism in the host response to intracellular bacterial pathogens during which microorganisms may undergo direct degradation in autophagolysosomes. As a highly virulent intracellular pathogen, *Francisella tularensis* has developed survival strategies to escape from the phagosome, replicate in the cytosol of mononuclear cells, and avoid degradation within the double-membrane vacuole during the autophagy-mediated response. The aim of this study was to investigate the role of the ATG5 autophagy protein in the host immune response to *Francisella tularensis* subsp. *holarctica*, live vaccine strain (LVS), since ATG5 plays an important role in autophagosome formation during canonical autophagy. In vitro experiments were conducted on immortalized bone marrow macrophages subjected to starvation-induced autophagy. Transgenic mice deficient in ATG5 of cells of the myeloid lineage (monocytes/macrophages and granulocytes) were used to analyze the immunological responses after intradermal infection. Cytokine levels were analyzed using Luminex, RT-qPCR, and ELISA, while inflammatory cell infiltration in the lung was analyzed by immunohistochemistry. Our results demonstrate that induced autophagy decreased bacterial replication in vitro. However, ATG5 deficiency in myeloid cells in vivo significantly diminished levels of pro-inflammatory cytokine IFN-γ in the sera, spleen, liver, and lung during *Francisella* infection. The attenuated pro-inflammatory response also led to significantly reduced macrophage and T cell infiltration in the lung tissue. Our findings also reveal that neutralization of IL-1β in myeloid ATG5ΔMye mice increased susceptibility to tularemia by increasing bacterial burden in organs.

## 1. Introduction

Autophagy is an evolutionarily conserved cellular mechanism, which involves the delivery of cytoplasmic substrates to lysosomes for degradation. It supports cellular homeostasis on the one hand, but, on the other, it may also promote the induction of diverse diseases [1]. Various factors can trigger the autophagy process, such as starvation, stress, DNA damage, damaged organelles, or intracellular pathogens [2]. The canonical pathway of macroautophagy includes the formation of a phagophore or isolation membrane, which precedes the formation of the autophagosome. The autophagosome is a double-membrane vesicle formed in the cytoplasm. It fuses with the lysosome, forming the autophagolysosome, and subsequent degradation of the unwanted content (organelles, proteins, or microorganisms) occurs in its lumen [1,3]. Formation of the phagophore is initiated at the phagophore assembly site (PAS) with the recruitment of autophagy-associated (ATG) proteins. To date, 41 ATGs have been identified [4], and ATG5 seems to have an indispensable role in autophagosome formation [5]. Conjugation of ATG5 to ATG12 and ATG16L1, forming the ATG12-ATG5/ATG16L1 complex, promotes autophagosome elongation and maturation [3]. Further, ATG5-ATG12 conjugate functions as a ubiquitin-protein ligase (E3) and participates in the lipidation of LC3 [1,4].

The role of autophagy in the degradation of microorganisms is called xenophagy [6]. Increasing evidence suggests that some pathogens evade degradation during autophagy, and for some of them, autophagy is beneficial. It has been suggested that autophagy plays a protective role against a *Mycobacterium tuberculosis* infection by direct elimination in autophagolysosomes [7,8]. *Listeria monocytogenes* [9] and *Shigella flexneri* [10] evade the autophagy process, while autophagy promotes intracellular replication of *Salmonella enterica* serovar Typhimurium [11].

To date, reports suggest that autophagy, as a cellular process, plays a critical role in the host’s innate and adaptive immune system. ATG5 is required for conventional dendritic cells (cDCs) to prime CD4+ T cells [12] and play an indispensable role in T cell survival [13].

Autophagy is involved in the development of an inflammatory response as well. It has been reported that a deficiency in autophagy genes induces the accumulation of the p62 protein [14] and the consequent activation of the NF-κb transcription factor [15]. ATG7 deficiency resulted in elevated IL-1β levels, but not TNF (tumor necrosis factor) and IL-6 levels during *Pseudomonas aeruginosa* sepsis [16]. *M. tuberculosis* infection of ATG5-deficient mice led to elevated IL-1α, IL-12, and IL-17 synthesis, but it did not influence the levels of IFN-γ and TNF [7].

*F. tularensis* is an intracellular pathogen capable of replicating within the cytosol of mononuclear cells. Macrophages have been described as key cells in *Francisella* replication and survival [17,18]. In the early stage of an infection, *Francisella* resides within a membrane-bound phagosome in macrophages before escaping into the cytosol [19,20,21]. After cytoplasmic replication, bacteria reenter autophagosomes while avoiding degradation within lysosomal compartments [22]. *Francisella* has evolved strategies to interfere with the mechanism of autophagy by downregulating autophagy-related genes (*beclin-1*, *Atg5*, *Atg12*, *Atg16L1*, *Atg7*, and *Atg4a*) and regulating autophagy signaling pathways (PI3K/Akt) [23]. It has also been reported that autophagy provides a nutrient source that supports *F. tularensis* replication [24].

The host response to *F. tularensis* is complex, requiring both innate and adaptive immunity. The innate response is induced by the interaction of *Francisella* components with membrane-bound Toll-like receptor 2 (TLR2) on macrophages. Triggering the TLR2 molecule induces pro-inflammatory gene expression and subsequent cytokine synthesis [25]. It has also been reported that *Francisella novicida* suppresses TLR-2-mediated pro-inflammatory cytokine secretion [26]. It has been reported that activation of IFN-γ in macrophages restricted bacterial proliferation, and that IFN-γ and TNF are essential in immune response to *F. tularensis* [27].

IL-1β processing to its active form requires inflammasome activation and the activity of the caspase-1 enzyme [28]. Autophagosomes degrade damaged mitochondria, reducing mitochondrial ROS (reactive oxygen species) and DNA that activate inflammasomes. Thereby, inhibition of autophagy activates the NLRP3 inflammasome [29] and increases IL-1α, IL-1β, and IL-18 release [30].

It has been reported that the ability of *Francisella* to escape from the phagosome is important for IL-1β processing and synthesis within human monocytes. Synthesis of IL-1β primarily depends on caspase-1 and the inflammasome adaptor protein ASC upon *F. tularensis* infection in vitro and in vivo [31]. It has also been documented that *F. tularensis* LVS strain suppresses early inflammasome activation and IL-1β secretion in the lung. The *FTL0325* gene is responsible for this suppression by interfering with NF-κB signaling [32].

In the present study, the strain *F. tularensis* subspecies *holarctica* LVS *holarctica* was used. LVS is attenuated for humans but virulent for mice. In the mouse model, LVS produces a well-characterized disease, sharing morphological features with human infection caused by virulent *F. tularensis* [19].

Our previously published results demonstrated an increased survival rate in ATG5ΔMye mice in comparison with controls upon infection. ATG5ΔMye mice also showed reduced bacterial replication in the lung, liver, and spleen. The histopathological changes in all three investigated organs were less severe in ATG5ΔMye mice than in controls [33].

However, an interplay between autophagy and the inflammatory response in vivo during *Francisella* infection has not been described. For this reason, we sought to investigate whether starvation-induced autophagy in vitro in mouse macrophages would impact bacterial replication. Next, we tested whether deletion of an autophagy gene, *Atg5*, in the myeloid lineage would influence the host inflammatory response in vivo during experimental tularemia. For that purpose, we used transgenic mice deficient in ATG5 in the myeloid lineage.

## 2. Materials and Methods

### 2.1. Bacterial Strain

*F. tularensis* subsp. *holarctica* strain LVS (Live Vaccine Strain) was provided by prof. Anders Sjöstedt (Umeå University, Umeå, Sweden). The strain was grown on gonococci agar base (Fischer Scientific, Pittsburgh, PA, USA) supplemented with IsoVitaleX (Fischer Scientific, Pittsburgh, PA, USA) at 37 °C with 5% CO_2_ for 48 h.

### 2.2. Cell Line

Immortalized mouse macrophages were cultivated on DMEM (Gibco, Thermo Fisher Scientific, Waltham, MA, USA) medium, with the addition of 10% FBS (Gibco, Thermo Fisher Scientific, Waltham, MA, USA) and 10% leukocyte colony-stimulating factor from L929 cells. For starvation purposes, cells were washed in Hank’s Balanced Salt Solution (HBSS, Capricorn Scientific, Ebsdorfergrund, Germany) and incubated in HBSS for six hours. Afterwards, 3-methyladenine (Sigma Aldrich, St. Louis, MO, USA) was added to stop the autophagy process.

### 2.3. Infection of the Cells

Control cells and cells with induced starvation were recovered in complete medium (DMEM) and infected with *Francisella tularensis* MOI 1:10. One hour after infection, cells were treated with gentamicin (5 mg/mL) (BioWhittaker, Lonza, Walkersville, MD, USA) for one hour to eliminate extracellular bacteria (time point 0).

At the indicated time points (24 h, 48 h, and 72 h), supernatant was collected for cytokine detection. To determine intracellular growth, cells were lysed using Saponin (Sigma Aldrich, St. Louis, MO, USA), and CFU was determined by plating bacteria on a Chocolate agar plate.

### 2.4. Mice

C57BL/6 Atg5^flox/flox^ mice (strain: B6.129S-Atg5<tm1Myok>) were purchased from RIKEN BioResource Research Center, Kyoto, Japan. Lys2cre mice (strain: B6.129P2-Lyz2^tm1(cre)/fo^/J) were purchased from the Jackson Laboratory, Bar Harbor, ME, USA. All animals were housed in the animal facility of the University of Rijeka, Faculty of Medicine, under specific pathogen-free conditions. Experiments were conducted according to the Institutional and National guidelines. The Atg5^flox/flox^ mice were crossed with mice homozygous for Lyz2<tm1(Cre)Ifo> to obtain myeloid cell-specific ATG5-deficient C57BL/6 Atg5^flox/flox^-Lyz-Cre mice (designated ATG5ΔMye). ATG5ΔMye mice were homozygous for the *loxP*- allele and heterozygous for the Cre transgene. C57BL/6 Atg5^flox/flox^ mice were used as controls. All experiments were conducted according to the regulatory procedures of the Republic of Croatia, respecting 3R method procedures. All experiments were approved by the Institutional Animal care and Use committee of the Faculty of Medicine, University of Rijeka, as well as by the Veterinary and Food Safety Directorate Croatia (Ministry of Agriculture).

### 2.5. Genotyping

The C57BL/6 Atg5^flox/flox^-Lyz-Cre mice genotypes were determined via PCR analysis of DNA to confirm that the *Atg5* fragment is deleted, as previously described [33,34]. The DNA of the C57BL/6 Atg5^flox/flox^ mice and/or Lys2cre mice was used as a positive control in the PCR analysis. To detect wild-type Atg*5* and Atg5^flox^ alleles, we used the following PCR primers: “ATG5 exon 3-1”, 5′-GAATATGAAGGCACACCCCTGAAATG-3′; “ATG5 check 2”, 5′-CAACGTCGAGCACAGCTGCGCAAGG-3′, “ATG5 short 2”, 5′-GTACTGCATAATGGTTTAACTCTTGC-3′; “oIMR3066”, 5′-CCCAGAAATGCCAGATTACG-3′; “oIMR3067”, 5′-CTTGGGCTGCCAGAATTTCTC-3′; “oIMR3068”, 5′-TTACAGTCGGCCAGGCTGAC-3′.

### 2.6. Infection Procedure

Six-to-eight-week-old male C57BL/6 Atg5^flox/flox^-Lyz-Cre and C57BL/6 Atg5^flox/flox^ mice were inoculated intradermally with the LVS strain at a dose of 5 × 10^4^ bacteria per mouse. Three mice per group were used in the whole experiment. Inoculum was confirmed by plating 10-fold serial dilutions on modified gonococci agar at 37 °C with 5% CO_2_ for 48 h. PBS solution was inoculated intraperitoneally into uninfected controls.

For the IL-1β depletion experiment, mice were inoculated intradermally (i. d.) with the LVS strain at a dose of 5 × 10^4^ bacteria per mouse. On days 0, 1, and 2 post-infection, 100 µg of anti- mouse IL-1β—neutralizing antibody (BioXCell, Lebanon, NH, USA) or Rat IgG isotype control antibody was inoculated intraperitoneally.

### 2.7. Immunohistochemistry

Samples for immunohistochemistry were taken 72 h after *F. tularensis* LVS i. d. infection from ATG5ΔMye and control mice. Three mice per group were used for a study. Mice were intracardially perfused with 0.9% NaCl in deep injection anesthesia/analgesia. Pulmonary vasculature was perfused with 10 mL of saline via the right ventricle. Following perfusion, the lungs of each group of infected mice were harvested aseptically, frozen in liquid nitrogen using Tissue Tek (Sakura Finetek Europe B.V, Alphen aan den Rijn, The Netherlands), and stored at −80 °C. Serial sections (10 µm) of tissue were analyzed by immunohistochemistry using a Vectastain Elite ABC Kit (Vector Laboratories, Burlingame, CA, USA) according to the manufacturer’s recommendations. In brief, to determine leukocyte and macrophage infiltration in the lungs, the respective rat anti-mouse monoclonal antibodies were used: CD4 antibody (Clone: H129.19, BD Biosciences, Rockville, MD, USA), CD8a (Clone: 53-6.7, BD Bioscience, Rockville, MD, USA), and F4/80 (Clone: A3-1, BioRad Laboratories, Hercules, CA, USA). Analyses were performed using an avidin-biotin complex technique with an appropriate biotinylated secondary antibody (Vectastain Elite ABC Kit, Vector Laboratories, Burlingame, CA, USA). The reaction was developed with DAB Peroxidase Substrate (Vector Laboratories, Burlingame, CA, USA) using 3,3′-diaminobenzidine as substrate and H_2_O_2_ as co-substrate. The sections were counterstained with hematoxylin, analyzed with the light microscope (Olympus IX51, Hamburg, Germany), and scanned with the Hamamatsu slide scanner (Hamamatsu S60, Herrsching am Ammersee, Germany). The observed changes have been assessed and performed at twenty random high-power fields (HPFs) to grade the severity of inflammation. Lung and liver tissue sections were investigated, and the semi-quantitative analysis was performed on the digitized slides for the infiltration of CD8+ T cells, CD8+ T cells, and F480+ macrophages (0—absent, 1—slight, 2—moderate, 3—severe). Liver and lung tissues were scored as absent if there was no infiltration of mononuclear cells. Involvement of the parenchyma was scored as slight when 25% of the total surface of the parenchyma was affected by inflammatory cells, moderate when 26–50% of the parenchyma was affected by inflammatory cells, and severe for involvement greater than 50%. The total histopathological result of each organ was calculated as the average of the results of individual criteria. The inflammation process and damaged regions were graded as described previously [34]. The total histopathology score of each organ was calculated as an average of individual criteria scores. The uninfected tissue was used as a baseline score.

### 2.8. RNA Extraction, Reverse Transcription, and Quantitative RT-PCR

RNA was extracted from cryopreserved organs using the Trizol reagent (Invitrogen, Carlsbad, CA, USA) according to the manufacturer’s instructions. RNA concentrations were measured fluorometrically. Reverse transcription to cDNA was performed using the QuantiTect Reverse Transcription Kit (Qiagen, Hilden, Germany) according to the manufacturer’s instructions. Expression of ATG5 and ATG7 RNA was measured using primers and SYBR Green Master Mix (Qiagen, Hilden, Germany). Cytokines were quantified using Master Mix (Applied Biosystems, Foster City, CA, USA) and TaqMan^®^ gene expression assays (Applied Biosystems, Foster City, CA, USA: IFN-γ (Mm01168134_m1), TNF-alpha (Mm00443258_m1), IL-10 (Mm00439614_m1), IL-1β (Mm01336189_m1). Samples were studied in triplicate. Data were normalized to murine TATA-box binding protein (TBP) and calibrated using the ΔΔCt method [35].

### 2.9. Cytokines and Chemokines Detection by Luminex and ELISA

Blood was taken from ATG5Δmye mice, control mice, and non-infected controls by an intracardiac puncture. After centrifugation, serum was removed, and levels of cytokines were determined using a commercial 23-plex kit (BioRad Laboratories, Hercules, CA, USA), according to the manufacturer’s instructions with a Bio-Plex 200 system (BioRad Laboratories, Hercules, CA, USA). ELISA assays for IFN-γ and for IL-10 in organs were performed using BD OptEIA Reagent Set A and B (BD Biosciences, Rockville, MD, USA). Standard stocks at different concentrations (31.3–4000 pg/mL) were taken as zero standard absorbance. The mean absorbance was calculated for each set of standards, controls, and samples, respectively. The mean zero standard absorbance was subtracted from each to obtain the final cytokine serum level (pg/mL).

### 2.10. Statistical Analysis

Statistical significances were determined using a two-tailed Student’s *t*-test, one-way ANOVA, Tukey’s multiple comparison test, and two-way ANOVA with Bonferroni posttest. Statistical analyses were performed using GraphPad Prism software version 5.01 for Windows (San Diego, CA, USA). In all cases, *p* < 0.05 was considered significant in comparison to the control mice.

## 3. Results

### 3.1. Induced Autophagy Reduced Intracellular Replication In Vitro

**Figure 1:**
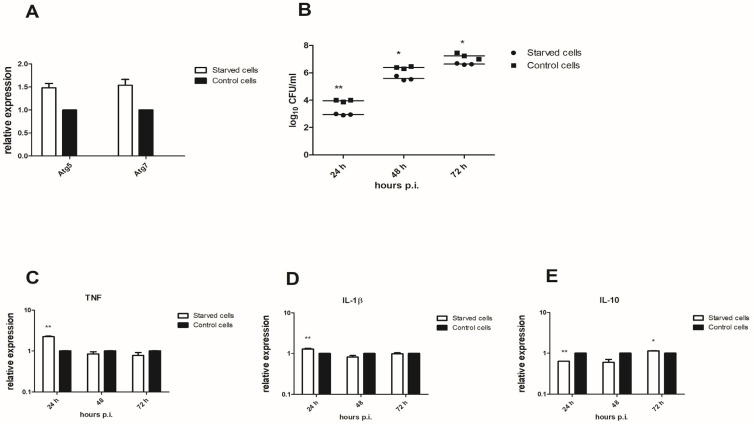
Growth kinetics (**B**) and cytokine expression (**C**–**E**) of the LVS-infected immortalized bone marrow macrophages, starved with HBSS and controls. For induction of starvation, cells were incubated in HBSS for 6 h, while the control group was incubated in RPMI. Induction of autophagy in starved cells was confirmed by the detection of Atg5 and Atg7 mRNA (**A**). Cells were infected with F. tularensis LVS at a dose of 1 × 10^7^ CFU/mL. One hour after infection, extracellular bacteria were removed with gentamicin. At indicated time points (24, 48, and 72 h p.i.), supernatant was collected for further RT-qPCR analyses of TNF (C), IL-1β (D), and IL-10 (E), and cells were lysed to determine intracellular bacterial load (**B**). The error bars represent the standard deviations of the triplicates. Statistical significances were determined using a two-tailed Student’s *t*-test and Welch’s correction. Statistically significant differences are indicated with an asterisk (* *p* < 0.05, ** *p* < 0.001).

To stimulate the starvation of the cells, immortalized mice bone marrow macrophages (iBMMs) were incubated in Hank’s buffered salt solution (HBSS) for 6 h, following 3-methyladenine (3-MA) treatment to inhibit the autophagic mechanism, as it has been previously described (27). Cells were then recovered in RPMI medium together with the control cells. To confirm the induction of autophagy in starved cells, expression of Atg5 and Atg7 mRNA was detected by RT-qPCR. Increased expression of Atg5 and Atg7 mRNA was observed in starved cells in comparison with the control 24 h after infection (Figure 1A).

Starved and control cells were further infected with *Francisella tularensis* LVS at an MOI of 1:10 to elucidate the impact of autophagy on intracellular replication and cytokine synthesis. Significantly reduced intracellular replication was detected in starved cells at all three indicated time points (24 h, *p* < 0.001; 48 h, *p* = 0.0150, and 72 h, *p* = 0.0450) post-infection (p.i.). (Figure 1B).

To evaluate the expression of pro-inflammatory cytokines in starved cells, mRNA levels of pro-inflammatory cytokines (IFN-γ, TNF, and IL-1β) and anti-inflammatory cytokine (IL-10) were measured by RT-qPCR. At 24 h p.i., IFN-γ expression was not detected in the starvation group nor in the control group. The expression of TNF was significantly higher in the starvation group in comparison to the control group (*p* = 0.0240) at 24 h p.i., as well as the expression of IL-1β (*p* = 0.0296). At 48 and 72 h p.i., pro-inflammatory cytokine levels decreased in starved cells and remained at the same level in the control group (Figure 1C,D). In contrast, starvation significantly decreased levels of IL-10 at 24 h p.i. when compared with controls (*p* = 0.0080). IL-10 levels continued to rise, and significantly higher levels were found compared to controls after 72 h (*p* = 0.0110) (Figure 1E).

### 3.2. ATG5 Impacts Pro-Inflammatory Response During F. tularensis LVS Infection

**Figure 2:**
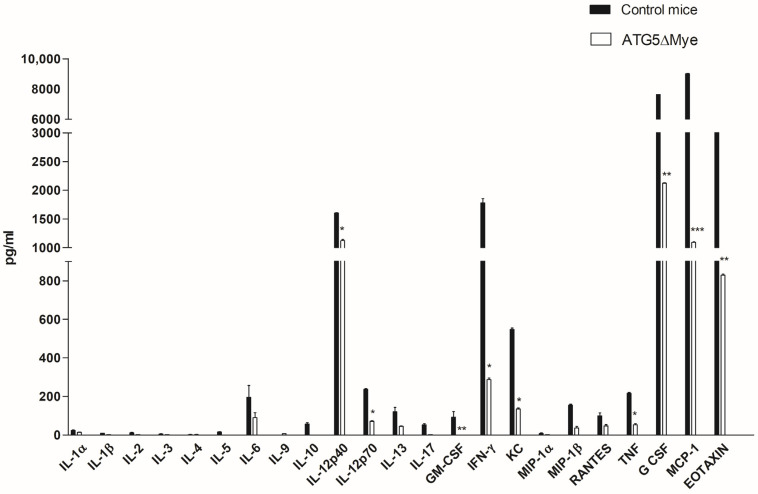
Systemic immunological responses in the control and ATG5ΔMye mice at 72 h p.i. 72 h upon intradermal infection with *F. tularensis* LVS, cytokine and chemokine levels in sera were determined by the Luminex assay. Significantly reduced serum levels of IL-12p40, IL-12p70, IFN-γ, KC, MIP-1b, TNF, G CSF, MCP-1, and Eotaxin in the ATG5ΔMye mice were observed in comparison to the control animals. Three mice per group were analyzed. The error bars represent the standard deviations of triplicate. Statistical significances were determined using a two-tailed Student’s *t*-test and Welch’s correction. Statistically significant differences are indicated with an asterisk (* *p* < 0.05, ** *p* < 0.01, *** *p* < 0.001).

To investigate the role of ATG5 in vivo, we used myeloid ATG5-deficient mice. The Atg5^flox/flox^ mice were crossed with mice homozygous for Lyz2<tm1(Cre)Ifo> to obtain myeloid cell-specific ATG5-deficient C57BL/6 Atg5^flox/flox^-Lyz-Cre mice (designated ATG5ΔMye). C57BL/6 Atg5^flox/flox^ mice were used as controls. Systemic inflammatory response was investigated in sera, measuring cytokine levels using a 20-plex mouse cytokine assay. ATG5ΔMye mice and control mice were infected intradermally with 5 × 10^4^ bacteria of the LVS strain. At 72 h p.i., sera were collected to determine levels of pro- and anti-inflammatory mediators using a 20-plex mouse cytokine Luminex assay. The absence of ATG5 in myeloid lineage significantly decreased levels of chemokines G-CSF (granulocyte colony-stimulating factor) (*p* = 0.0012), MCP-1 (monocyte chemoattractant protein-1) (*p* = 0.0009), EOTAXIN (CCL11) (*p* = 0.0013), and KC (keratinocyte-derived chemokine) (*p* = 0.0139) (Figure 2). In addition, statistically significant decreases in IFN-γ (*p* = 0.0317), TNF (*p* = 0.0302), IL-12p40 (*p* = 0.0317), and IL-12p70 (*p* = 0.0204) levels were observed in the ATG5ΔMye mice in comparison with the controls (Figure 2). In conclusion, myeloid ATG5 deficiency impaired the production of chemokines and pro-inflammatory cytokines during *F. tularensis* infection. Impaired production of cytokines and chemokines correlates with our previous results on decreased bacterial burden in ATG5ΔMye mice in comparison with the controls (Supplemental Material Figure S1).

### 3.3. ATG5 Altered Immune Response During Experimental Tularemia

**Figure 3:**
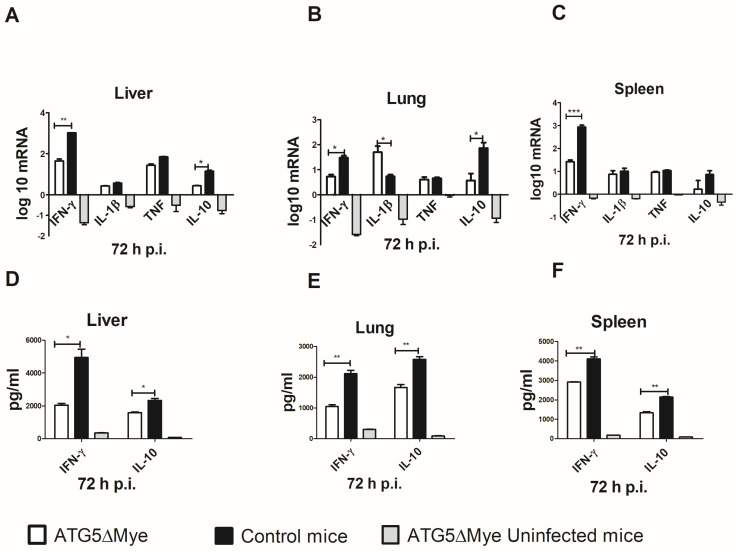
The synthesis of cytokines IFN-γ, TNF, IL-10, and IL-1β in liver, lung, and spleen in the control and ATG5ΔMye mice during *F. tularensis* LVS infection. The mRNA levels of IFN-γ, TNF, IL-10, and IL-1β were measured 72 h p.i. using RT-qPCR. The secretion of IFN-γ and IL-10 was analyzed by ELISA. Reduced expression of IFN-γ and IL-10 in all three investigated organs of ATG5ΔMye mice was detected when compared with control mice (**A**–**C**). In the lung of ATG5ΔMye mice, increased mRNA levels of IL-1β were observed. Reduced synthesis of IFN-γ and IL-10 in the liver, lung, and spleen was further confirmed by ELISA (**D**–**F**). ATG5ΔMye mice served as uninfected controls. The error bars represent the standard deviations of triplicate samples. Statistical significance was determined using one-way ANOVA and Tukey’s multiple comparison test. * *p* < 0.05 was considered significant in comparison to control mice. Statistically significant differences are indicated with an asterisk (* *p* < 0.05, ** *p* < 0.01, *** *p* < 0.001). To confirm the obtained RT-qPCR results, pro-inflammatory and anti-inflammatory cytokine production was investigated by ELISA. Significantly decreased levels of IFN-γ were detected in the liver (*p* < 0.05), lung (*p* < 0.01), and spleen (*p* < 0.01) of ATG5ΔMye mice, in comparison with the control group at 72 h. p.i. IL-10 synthesis was also significantly reduced in the liver (*p* < 0.05), lung (*p* < 0.01), and spleen (*p* < 0.01) of ATG5ΔMye mice (**D**–**F**). Thereby, protein levels of IFN-γ and IL-10 confirmed the results of transcriptional findings.

Our previous results had shown that myeloid ATG5 deficiency impaired *Francisella* growth in the liver, spleen, and lung in comparison to littermate controls (Supplemental Material Figure S1) [33]. To evaluate the impact of myeloid ATG5 on the cytokine response in the mouse model of *Francisella* infection, mRNA levels of IFN-γ, TNF, IL-10, and IL-1β were measured by RT-qPCR in the liver, lung, and spleen. At 72 h p.i., ATG5ΔMye mice significantly suppressed production of IFN-γ in the liver (*p* < 0.01), lung (*p* < 0.05), and spleen (*p* < 0.001) in comparison to controls. IL-10 levels were also significantly reduced in the liver (*p* < 0.05) and lung (*p* < 0.05) of ATG5ΔMye mice (Figure 3A–C). In contrast, ATG5ΔMye mice demonstrated statistically higher mRNA IL-1β levels in the lung when compared to the control littermates (*p* < 0.05) (Figure 3B).

**Figure 4:**
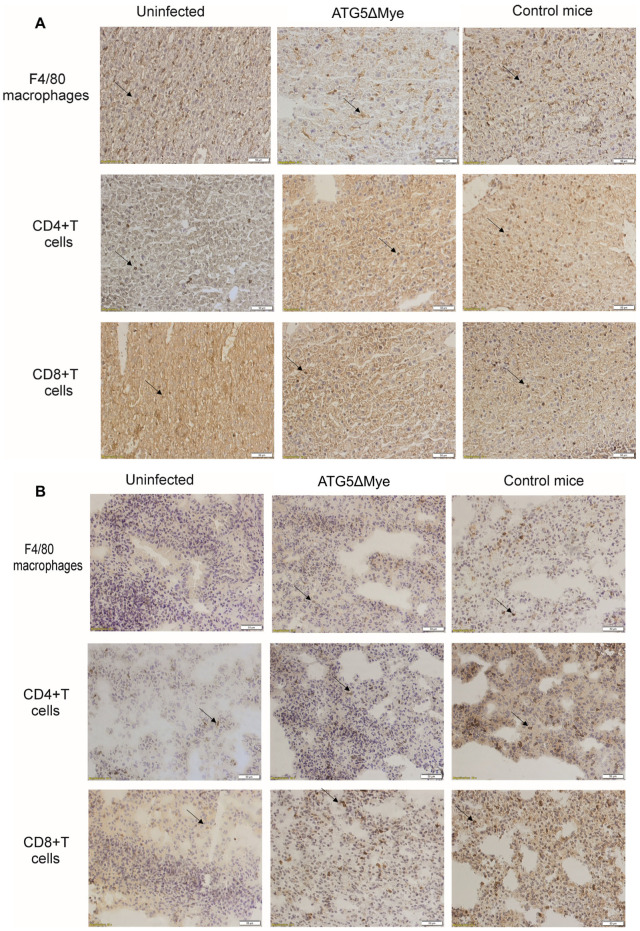

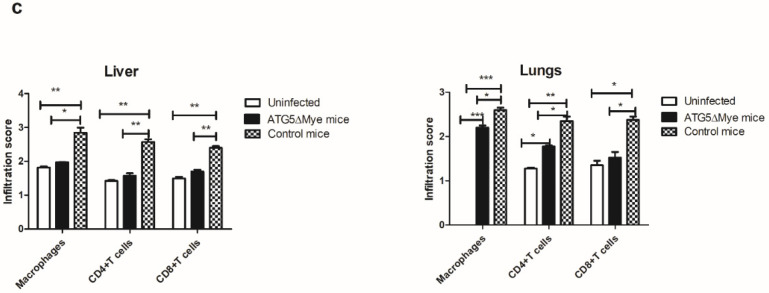
Immunohistochemical staining of F4/80 macrophages, CD4+ T cells, and CD8+ T cells in the liver and lung of ATG5ΔMye, control, and uninfected control mice during *F. tularensis* LVS infection. 72 h after infection of mice with *F. tularensis* LVS, liver and lung sections were analyzed by immunohistochemistry to detect F4/80+ macrophages, CD4+ T cells, and CD8+ T cells (**A**–**C**). Samples were microscopically examined under 20× magnification; the scale bar indicates 50 µm. Statistical significance was determined using one-way ANOVA and Tukey’s multiple comparison test. * *p* < 0.05 was considered significant in comparison to control mice. Statistically significant differences are indicated with an asterisk (* *p* < 0.05, ** *p* < 0.01, *** *p* < 0.001).

Finally, considering that T cells are important in controlling *Francisella* growth within macrophages [36] and that macrophages and neutrophils were previously identified as primary cell types in the lung upon *Francisella* intradermal inoculation [37], we sought to determine the abundance of the T cell subsets and macrophages in the liver and lung. ATG5ΔMye and control mice were infected intradermally with 5 × 10^4^ bacteria of the LVS strain per mouse. C57BL/6 Atg5^flox/flox^ mice were used as uninfected controls. PBS was injected into uninfected controls instead of bacteria. Immunohistochemical staining of the liver and lung sections was examined by light microscopy. At 72 h p.i., the presence of macrophages, CD4+ T cells, and CD8+ T cells was analyzed. Prominent CD4+ T and CD8+ T lymphocyte infiltrations were detected in the liver (Figure 4A) and lung tissue (Figure 4B) of both ATG5ΔMye and control mice. Less severe infiltration of CD4+ (*p* < 0.01) cells and CD8+T (*p* < 0.01) cells was observed in the liver sections of ATG5ΔMye mice when compared to the controls (Figure 4C). Significant lower infiltration of CD4+ (*p* < 0.05) cells and CD8+T (*p* < 0.05) cells was observed in the lung sections of ATG5ΔMye mice when compared to the controls as well (Figure 4C). Moreover, aggregates of macrophages were abundantly present in liver (Figure 4A,C) and lung (Figure 4B,C) tissue sections of both ATG5ΔMye and control animals, although ATG5ΔMye mice were found to have less severe macrophage density in liver and lung (*p* < 0.05) (Figure 4C).

### 3.4. IL-1β Neutralization Increased Susceptibility to F. tularensis LVS Infection in ATG5ΔMye, but Not in Control Littermates

**Figure 5:**
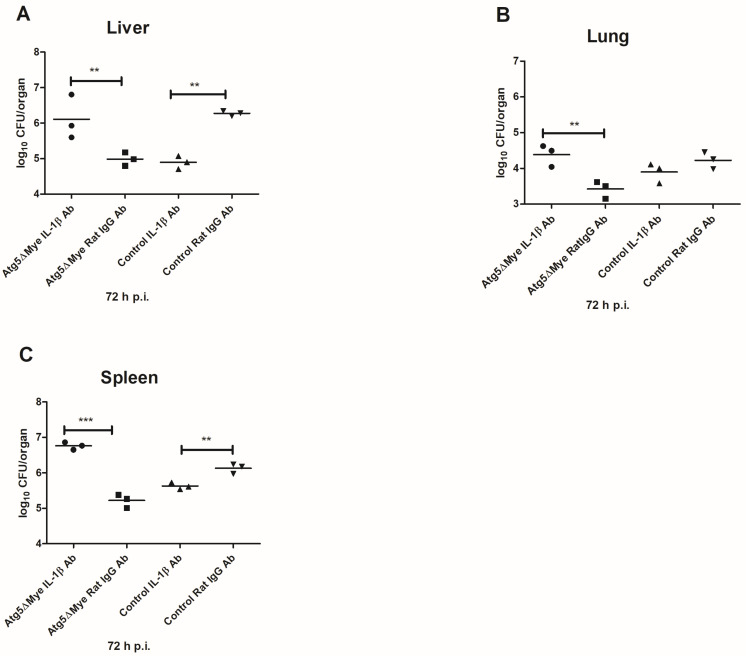
Growth kinetics of the LVS strain in the spleen, liver, and lung of the ATG5ΔMye mice and control mice treated with IL-1β antibody or control Rat IgG antibody. Mice were infected intradermally with 5 × 10^4^ bacteria per mouse. Each group of mice (ATG5ΔMye and control group) was treated with IL-1β antibody or control Rat IgG antibody. To determine the bacterial loads in the liver (**A**), lung (**B**), and spleen (**C**), organs were harvested and homogenized. Three mice per group were used in this experiment. Mean values are indicated. Statistical significance was determined using a two-way ANOVA with Bonferroni post-test. Statistically significant differences are indicated with an asterisk (** *p* < 0.01, *** *p* < 0.001).

Next, we sought to investigate the role of IL-1β in experimental tularemia during myeloid ATG5 deficiency. To investigate the impact of IL-1β on cytokine secretion, ATG5ΔMye mice and control mice were infected with *F. tularensis* LVS, with a dose of 5 × 10^4^ bacteria. Administration of neutralizing IL-1β antibody, or isotype control antibody (Rat IgG), was performed at days 0, 1, and 2 p.i. At time point 72 h p.i., the bacterial counts in the liver, lung, and spleen were determined. There was a statistically significant increase in the bacterial burden in the liver (*p* < 0.01), lung (*p* < 0.01), and spleen (*p* < 0.001) of ATG5ΔMye mice treated with neutralizing IL-1β antibody than in those treated with control antibody (Figure 5). In contrast, treatment with IL-1β antibody significantly decreased bacterial load in the liver (*p* < 0.01) and spleen (*p* < 0.01) of control mice, but the bacterial load in the lung was comparable (*p* = 0.1958).

**Figure 6:**
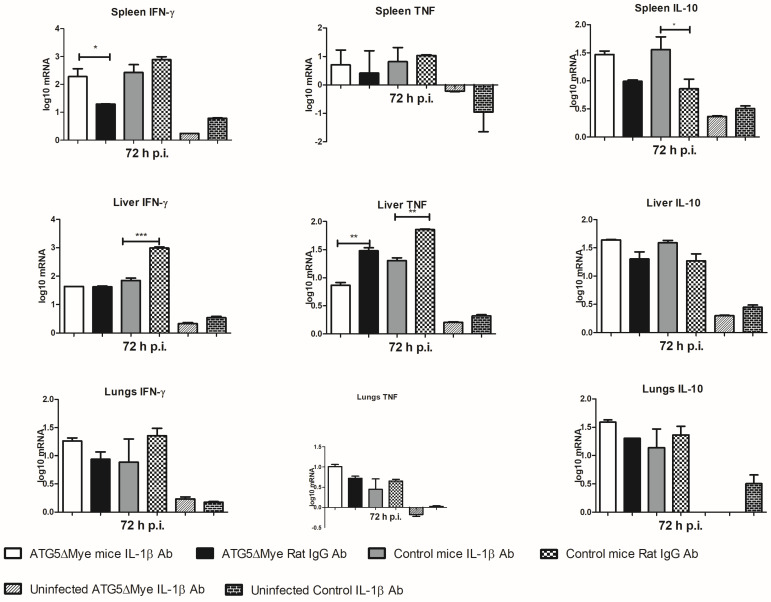
The synthesis of cytokines IFN-γ, TNF, and IL-10in the liver, lung, and spleen in the ATG5ΔMye mice and control mice during *F. tularensis* LVS infection. IL-1β antibody or control Rat IgG antibody was administered to *F. tularensis* LVS-infected mice. 72 h after infection, mRNA levels of IFN-γ, TNF, and IL-10 were measured using RT-qPCR. Three mice per group were analyzed in this experiment. The error bars represent standard deviations of triplicate samples. Statistical significance was determined using one-way ANOVA and Tukey’s multiple comparison test. Statistically significant differences are indicated with an asterisk (* *p* < 0.05, ** *p* < 0.01, *** *p* < 0.001).

**Figure 7:**
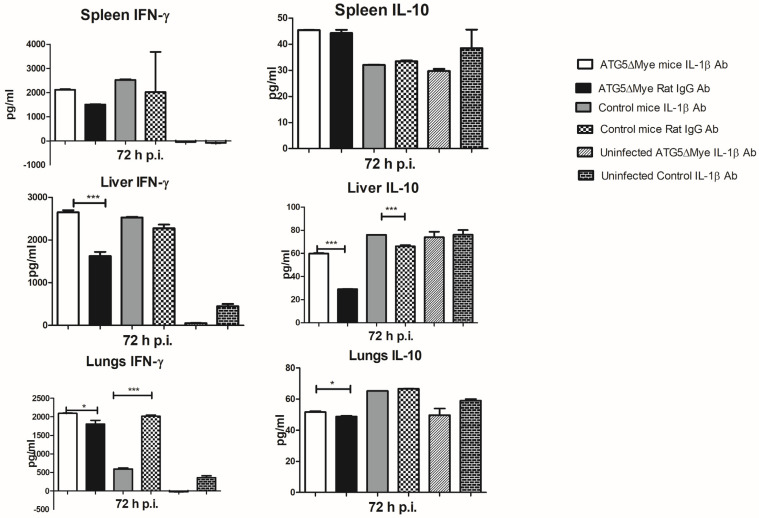
The synthesis of cytokines IFN-γ, and IL-10 in the liver, lung, and spleen in the ATG5ΔMye mice and control mice during *F. tularensis* LVS infection. IL-1β antibody or control Rat IgG antibody were administered to *F. tularensis* LVS-infected mice. 72 h upon infection, synthesis of IFN-γ and IL-10 cytokines was analyzed by ELISA. Three mice per group were analyzed in this experiment. The error bars represent standard deviations of triplicate samples. Statistical significances were determined using one-way ANOVA and Tukey’s multiple comparison test. Statistically significant differences are indicated with an asterisk (* *p* < 0.05, *** *p* < 0.001).

Next, to evaluate the effect of IL-1β attenuation on cytokine response during experimental tularemia, mRNA levels of IFN-γ, TNF, and IL-10 in the liver, lung, and spleen of ATG5ΔMye and control mice were measured by RT-qPCR. IFN-γ and IL-10 were also detected by ELISA. At 72 h p.i., ATG5ΔMye mice treated with IL-1β antibody revealed a significant increase in IFN-γ expression in the spleen (*p* < 0.05) and TNF (*p* < 0.01) in the liver in comparison with the same group treated with Rat IgG antibody. At the indicated time point, control mice treated with IL-1β antibody showed significantly decreased liver IFN-γ (*p* < 0.001) and TNF expression (*p* < 0.01) in comparison with the same group treated with Rat IgG antibody (Figure 6). ELISA results revealed statistically significant increased IFN-γ and IL-10 production in the liver and lung (but not in the spleen) of ATG5ΔMye mice treated with IL-1β antibody in comparison with the same group treated with Rat IgG antibody. In addition, in control mice treated with IL-1β antibody, significantly decreased IFN-γ secretion was detected only in the lung (*p* < 0.001), but increased IL-10 secretion was detected in the liver (*p* < 0.001) (Figure 7).

## 4. Discussion

In this work, we have attempted to define the importance of autophagy and the ATG5 protein in the pathogenesis of *Francisella* infection in vivo. The importance of autophagosomes/autolysosomes in the clearance of intracellular bacteria has been described for a variety of intracellular pathogens. Autophagy has been found to play an important role in the control of *M. tuberculosis* infection [38]. Mice with ATG5-deficient macrophages also showed increased *T. gondii* infection [39]. But there are contradictory data about the interaction of *Francisella* and the autophagy protein ATG5.

Some reports suggest that *Francisella* is able to delay the autophagy response. One of the mechanisms is the downregulation of several autophagy genes, including *Atg*5, in the early phase of infection. The other mechanism is downregulation of signaling pathways, such as phosphatidylinositol-3-kinase/protein kinase B (PI3K/Akt) signaling and Toll-like receptor signaling. This results in a reduced autophagy response and lower cytokine production [23]. Steele et al. reported that autophagy provides amino acids required for the *F. tularensis* growth and that inhibition of autophagy decreased the growth of the *F. tularensis* Schu S4 strain in human macrophages. The same authors stated that ATG5 is not required for effective intracellular growth of *Francisella* [24]. On the other hand, our previous results have shown that the presence of ATG5 directs *F. tularensis* LVS to the autophagic vacuole, which promotes its proliferation [33]. Although the bacterium is able to invade and proliferate in many cell types [40], macrophages play an indispensable role in the pathogenesis of *Francisella* infection. Our in vitro results demonstrate that starvation- induced autophagy increases the expression of *Atg5* and *Atg7* mRNA in mouse immortalized macrophages. Interestingly, induction of autophagy suppressed the intracellular proliferation of bacteria but increased pro-inflammatory response. In concordance with our results, Dupont et al. reported that the stimulation of autophagy by starvation promoted IL-1β secretion in vitro in an ATG5-dependent manner [41]. To determine how ATG5 protein affects the course of experimental tularemia in vivo, we used a mouse model of Atg5^flox/flox^-Lyz-Cre with macrophage and granulocyte-specific deletion of the *Atg5* gene [34]. Since the synthesis of neutrophil-recruiting chemokines is an important step in an immune response, we tested the levels of chemokines in the sera of ATG5ΔMye mice and compared them to the control group. The levels of G CSF, KC, MCP-1, EOTAXIN, and MIP-1β chemokines were significantly reduced in ATG5ΔMye mice in comparison with controls, highlighting an aberrant activation of the immune response during ATG5 deficiency.

The next step involved assessing pro-inflammatory cytokine secretion, as IFN-γ and TNF are critical for controlling *Francisella* LVS infection [42]. Macrophages activated with IFN-γ restricted the intracellular growth of *F. tularensis* LVS [43] and *F. tularensis* Schu strain [27]. Our results revealed that IFN-γ levels were significantly reduced during myeloid ATG5 deficiency, at the peak of the disease, suggesting an attenuated pro-inflammatory response due to impaired autophagy. Reduced TNF levels in ATG5ΔMye mice support this hypothesis. To date, reports about the influence of ATG5 on IFN-γ are contradictory. ATG5 deficiency in mouse embryonic fibroblasts inhibited IFN-γ-induced pro-inflammatory response [44]. In contrast, the deficiency of ATG5 in macrophages and granulocytes did not diminish IFN-γ production in vivo upon infection with *Toxoplasma gondii* [39].

Another important pro-inflammatory cytokine in the course of *Francisella* infection is IL-1β [45]. However, the interplay between autophagy and IL-1β secretion is complex, and there are contradictory reports on whether autophagy reduces or increases secretion of IL-1β. IL-1β stimulates autophagy, which is of high relevance for *M. tuberculosis* control and elimination [46]. Deficiency of ATG7 protein increased IL-1β levels during sepsis caused by *Pseudomonas aeruginosa* infection [47]. Myeloid ATG5-deficient mice had increased serum levels of IL-1β due to the elevated hepatic caspase-1 activation [48]. Saitoh et al. reported that ATG16L1-deficient macrophages produce high amounts of the inflammatory cytokines IL-1β and IL-18 after TLR4 LPS stimulation [49]. Autophagy also promotes IL-1β secretion in neutrophils [50]. Our findings provide evidence that ATG5 deletion in myeloid cells increased IL-1β mRNA levels in the lung of mice upon i.d. infection with *F. tularensis* LVS when compared to the control animals. However, IL-1β levels in the liver, spleen, and sera were not affected by ATG5ΔMye and were comparable to or even slightly lower (liver, spleen) than in a control. This discrepancy in the results may be explained by the fact that lung tissue macrophages are important in IL-1β synthesis [51], and interstitial macrophages, together with the neutrophils, are the dominant infected cells in the lung after intradermal inoculation of *Francisella* [37].

IL-10 has been identified as a key cytokine in the pathogenesis of *F. tularensis* LVS infection by regulating the balance between immune response and progression of the disease. Reported data suggest that IL-10 is secreted during the early stage of *F. tularensis* LVS pulmonary infection by lung dendritic cells [52]. Our data reveal significantly higher IL-10 levels in the liver, lung, and spleen of control mice at 72 h p.i. than in ATG5ΔMye mice, suggesting a positive correlation between autophagy and IL-10 secretion. As IL-10 inhibits caspase-1-dependent inflammasome activation [53], lack of IL-10 may thereby trigger caspase-1-dependent IL-1β secretion in ATG5-deficient mice.

Moreover, ATG5 is required for CD8+ and CD4+ T cell survival [13]. In this context, we have proposed that a deficiency of ATG5 in myeloid cells would also diminish the appropriate T cell response, as T cells were determined to have an important role in the control of *Francisella* intracellular growth [54]. The protective role of T cells is primarily achieved through IFN-γ and TNF, which are critical for controlling *Francisella* LVS infection [42]. Nevertheless, aerosol LVS infection increased CD4+ T cell number in the lung [36]. Our findings demonstrate slightly reduced infiltration of F4/80+ macrophages, CD4+ T cells, and CD8+ T cell subsets in the liver and lung of ATG5ΔMye and LVS-infected mice. Other published reports also reveal that autophagy impacts T cell development and function. The deficiency of autophagy in lymphoid progenitors reduced the number of CD4+ and CD8+ T cells, with a more severe reduction in CD8+ T cells [13]. In addition, it has been reported that alveolar macrophages were the predominant infected cell type in the lung of LVS-infected mice 1 day after infection. While the number of alveolar macrophages remained unchanged up to day 3 post-infection, the number of CD11b^high^ macrophages increased from day 1 to day 3 [55].

It follows from the above that autophagy and IL-1β play an important role in controlling bacterial infections. A protective role of IL-1β during pulmonary infection has been described, and IL-1β recruits and activates polymorphonuclear cells and macrophages [56]. Autophagy has been reported to block IL-1β-induced hepatocyte death and inflammation [57]. Blocking the IL-1 receptor inhibited inflammasome activation, restored autophagy, and decreased IL-1β secretion in mice with chronic granulomatous disease [58]. Furthermore, the natural regulation between autophagy and inflammasome activation is a key process in IL-1β secretion and inflammation.

To understand how IL-1β neutralization impacts *Francisella* replication and course of disease, we investigated the interplay between ATG5 and IL-1β in vivo. Indeed, our findings suggest that neutralizing IL-1β in ATG5ΔMye mice significantly increased bacterial load in organs and the production of the cytokines IFN-γ and IL-10 compared with ATG5ΔMye mice treated with a Rat IgG control antibody. Treatment of control mice with an IL-1β antibody decreased bacterial burden in organs, as well as IFN-γ and TNF levels, compared with controls treated with a Rat IgG control antibody. Our findings demonstrate that attenuation of IL-1β, together with ATG5 deficiency in myeloid cells, increases susceptibility to tularemia. Although various data report the beneficial role of IL-1β in suppressing bacterial replication [59,60], results reported by Bawazeer et al. indicate that IL-1β inhibition reduces the severity of influenza in vivo [61].

Other reported data on *Il-1β*^−/−^ and *Il-1r1*^−/−^ knockout mice suggest that these animals were more susceptible to lung infection induced with *F. tularensis* LVS than C57BL/6 controls due to decreased IgM production [59].

In summary, the presented study highlights the role of ATG5 in regulating the immune response throughout the course of *F. tularensis* LVS infection. Although starvation-induced autophagy did not impact *Francisella* replication in vitro, the presented data suggest that ATG5 is required for the effective replication of *Francisella tularensis*, LVS strain in cells of myeloid origin in vivo. The presented data also suggest that ATG5 is required for the appropriate regulation of an adaptive immune response and synthesis of pro-inflammatory cytokines.

However, the impact of autophagy on *Francisella* infection cannot be generalized. This work reveals differences in the in vivo and in vitro models of *Francisella* infection. Next, the impact of autophagy cannot be extended to the other strains, primarily to the pathogenic *F. tularensis* SchuS4 strain (Type A). First of all, differences in the genome structure between Type A and Type B strains and the level of genomic rearrangements between them may affect their pathogenicity [60].

Although the LVS strain is pathogenic for the mice, a rapid disease progression, increased pathological changes in organs, and upregulation of the inflammatory genes and repressed expression of pro-apoptotic and anti-apoptotic genes were observed in the SchuS4-infected mouse model. All the above-mentioned may affect intracellular processes, including autophagy. It has also been reported that the SchuS4 strain avoids ubiquitination steps, essential for autophagic recognition [61]. Thereby, the influence of ATG5 on the course of *Francisella* infection and subsequent immunological response may differ and may depend on the pathogen and model of infection.

## 5. Conclusions

The presented data suggest that ATG5 is required for the effective replication of *Francisella tularensis*, LVS strain, in cells of myeloid origin in vivo but not in vitro. ATG5 is also required for the subsequent regulation of an adaptive immune response and cytokine synthesis. Moreover, our findings reveal that attenuation of IL-1β, together with ATG5 deficiency in myeloid cells, increased susceptibility to tularemia.

## Data Availability

The original contributions presented in this study are included in the article. Further inquiries can be directed to the corresponding author.

## Supplementary Materials

The following supporting information can be downloaded at: https://www.mdpi.com/article/10.3390/microorganisms14071593/s1, Figure S1. Growth kinetics of LVS in the spleen, liver, and lung of ATG5ΔMye and control mice.
