# Supplementary figures and images for: Modulatory Role of ATG5 Protein in Immune Modulation During Experimental Tularemia
Source: Microorganisms. 2026 Jul 21;14(7):1593. doi: 10.3390/microorganisms14071593 (PMC13414373; doi:10.3390/microorganisms14071593)

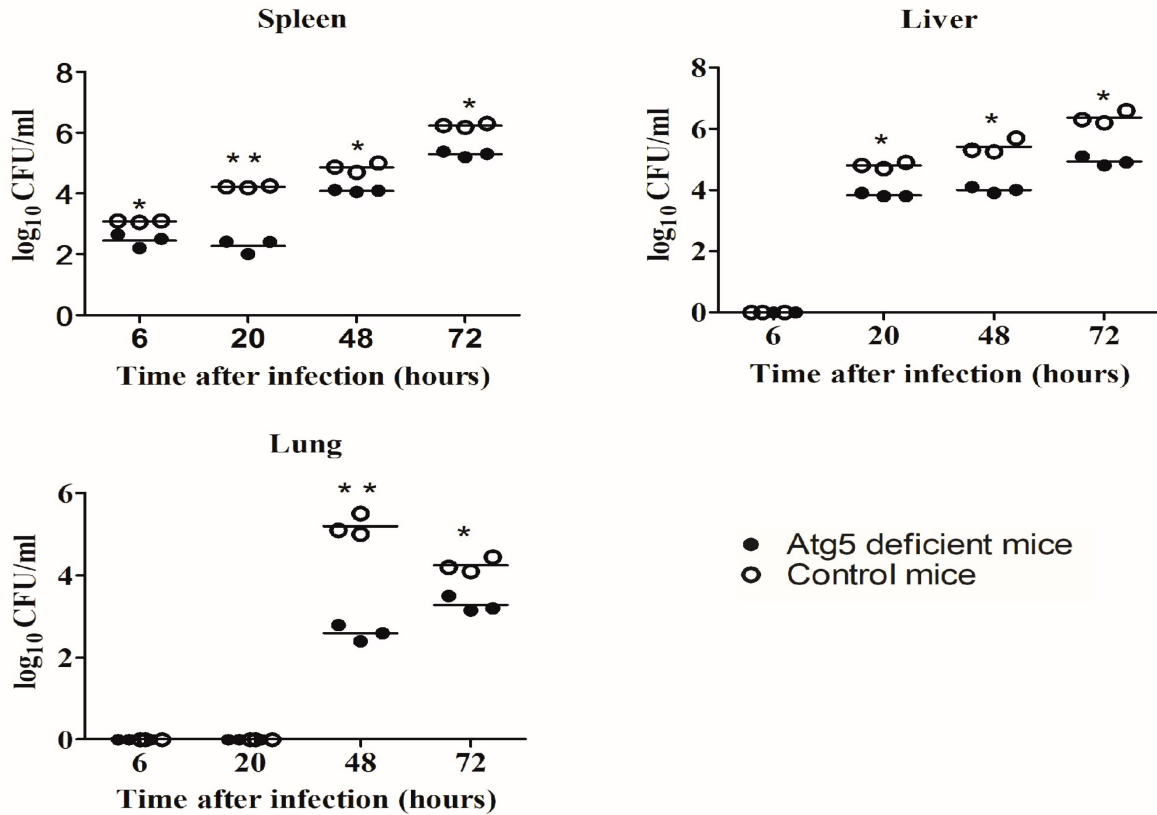

**Figure S1.** Growth kinetics of LVS in the spleen, liver, and lung of ATG5ΔMye and control mice.

Supplement: Supplementary file 1 [file microorganisms-14-01593-s001.zip › microorganisms-4331075-supplementary.pdf]
